# The muscle nutritional components analysis of golden pompano (*Trachinotus blochii*) in different mariculture area, growth stages, and genders

**DOI:** 10.3389/fnut.2023.1148687

**Published:** 2023-08-04

**Authors:** Liping Shi, Feibiao Song, Shuiyao Xing, Weiwei Zhang, Yesong Liang, Kaixi Zhang, Junlong Sun, Jian Luo

**Affiliations:** Sanya Nanfan Research Institute, State Key Laboratory of Marine Resource Utilization in South China Sea, Hainan Aquaculture Breeding Engineering Research Center, Hainan Academician Team Innovation Center, Hainan University, Haikou, China

**Keywords:** *Trachinotus blochii*, amino acids, fatty acids, aquaculture industry, growth indices, food supply, mariculture practices

## Abstract

Golden pompano (*Trachinotus blochii*) is an economically important fish which exhibits sexual size dimorphism and is widely cultivated in the southern seas of China. To evaluate the nutritional composition of *T. blochii* of different mariculture areas, growth stages, and genders, the moisture, ash, amino acids, and fatty acids in the muscle were measured using national standard biochemical assay. The analysis found 16 kinds of amino acids in the muscle of *T. blochii*. The EAA contents of fish from Guangdong (GD) and Guangxi (GX) were significantly lower than those of Hainan (HN) and Fujian (FJ) (*p* < 0.05). The unsaturated fatty acids were higher in *T. blochii* cultured in HN and FJ (*p* < 0.05). Within the same sea area, the contents of TAA, EAA, DAA, and PUFA increased with growth in *T. blochii*, but the differences were not significant (*p* > 0.05). EAA/TAA and EAA/NEAA conformed to the ideal FAO/WHO model. The AAS, CS, and EAAI scores of amino acids within groups gradually increased with growth. The TAA, EAA and PUFA contents in females were higher than in males (*p* > 0.05). The slightly higher amounts of amino acids and fatty acids in female *T. blochii* indicated females had higher nutritional value. In conclusion, the HN and FJ groups, the later growth stages, and the female *T. blochii* had generally higher nutritional values than their respective counterparts. These results provide fundamental data supporting all-female *T. blochii* breeding and culture, and optimized marketing body size.

## 1. Introduction

The rapid development of aquaculture has important implications for human food supply and security, as well as for economic growth and environmental sustainability (1). Fish are a great source of proteins, vitamins, essential minerals, and unsaturated fatty acids (2). *Trachinotus blochii* is an omnivorous fish (3) which is loved by consumers for its tender meat, delicious taste, and high nutritional value (4). Naturally, *T. blochii* is widely distributed in China, Southeast Asia, Australia, Japan, Eastern Africa, and the Atlantic Ocean (5) and highly valued in Southeast Asian and North American markets (6). Therefore, *T. blochii* has high economic value and broad marketability. After years of development, *T. blochii* has become the second largest maricultured fish in China (7) and, as such, has been heavily researched by scholars as a hot research topic in recent years.

The nutritional quality of aquatic products like fish mainly depend on their amino acids and fatty acids, which are influenced by their size and physiological status and the feed they are supplied. Differences in the mariculture environment and feed have been shown to have the greatest influence on nutritional quality (8). Different feeds and feeding regimes can influence meat quality, taste, and composition of cultured fish (9). Research on grass carp (*Ctenopharyngodon idella*) raised under different cultivation methods showed that the nutritional quality in the mono-culture group was better than that in mixed-culture group (10). Huang et al. (11) also analyzed the muscle nutrients of *Aristichthys nobilis* reared in different waters and showed that *A. nobilis* reared in waters with a large surface area were superior to waters with a small surface area in terms of nutrient content, meat content, and taste. Studies on *Culter alburnus* (12) and *Pelteobagrus fulcidraco* (13, 14) observed that the nutritional composition and quality of fish was affected by culture area, growth stage, and gender. The existing research on *T. blochii* has focused on fish seed cultivation, disease prevention, feed formula, and breeding technology. However, the nutritional quality of the muscle of *T. blochii* in different mariculture areas, growth stages, and genders still unclear. To provide basic theoretical guidance for *T. blochii* culture, we investigated how muscle nutrients and quality vary in *T. blochii*.

In this study, we investigated *T. blochii* of different mariculture areas, growth stages, and genders using biochemical methods to conduct comparisons of muscle moisture, ash, amino acids, and fatty acids. This study will provide basic reference data that will help consumers better understand the nutritional value of *T. blochii* and help in the rational development and utilization of *T. blochii* resources and optimization of artificial cultivation methods.

## 2. Materials and methods

### 2.1. Sample collection and preparation

**Figure 1:**
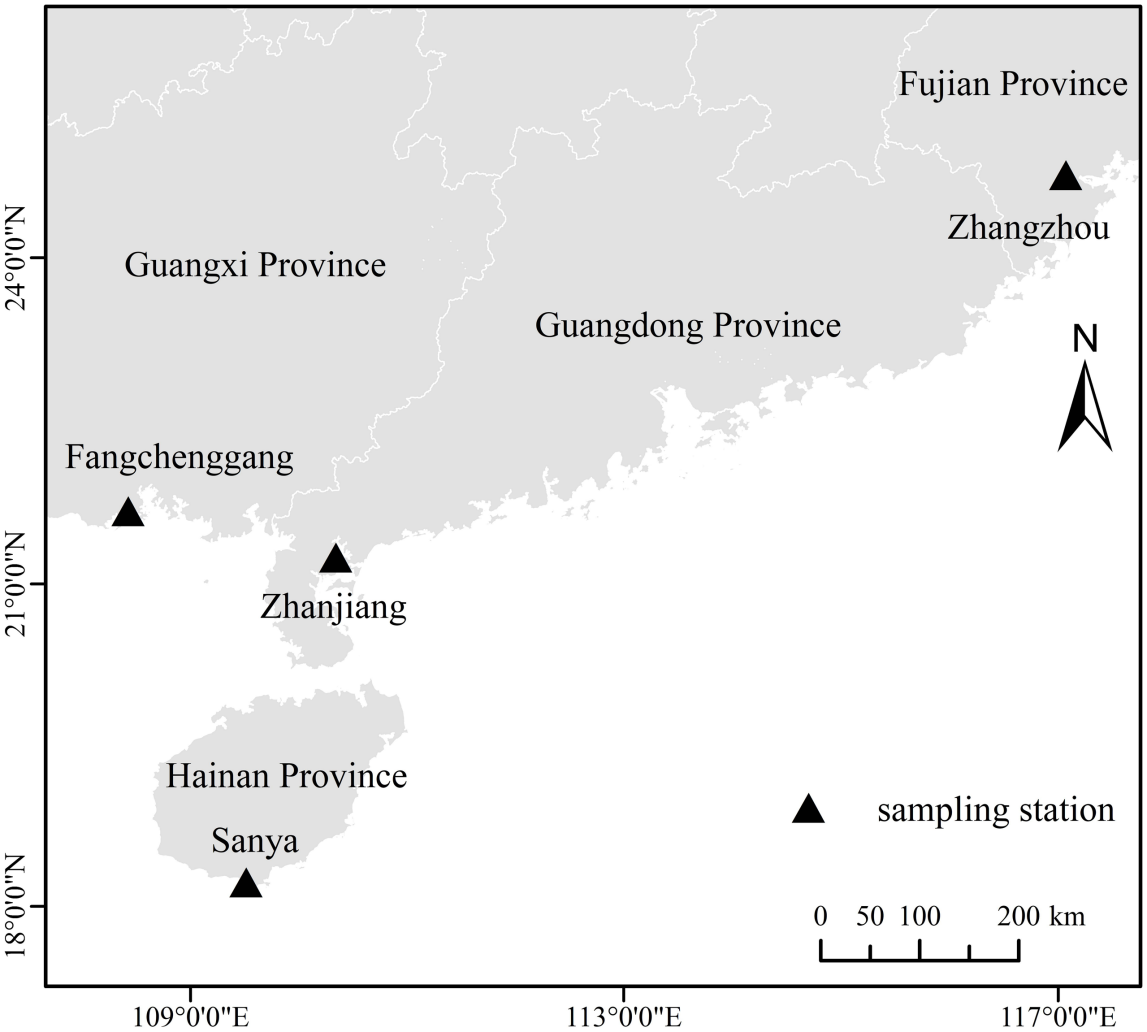
Sampling sites of the 4 cultured groups of *T. blochii.*

The fish were released into the sea cage of 4 breeding bases in Sanya of Hainan, Zhanjiang of Guangdong, Fangchenggang of Guangxi, and Zhangzhou of Fujian in April and fed identical diets (Yuehai Feed, Guangdong Yuehai Feed Group). The sources of seedlings in these four areas are same, they all came from Blue Grain Technology Co., Ltd. (Sanya, Hainan Province, China). Thirty *T. blochii* samples with body weights of 500.74 ± 20.09 g were collected from each of the four areas as experimental materials in September 2021. The experiment was divided into four groups, Hainan (HN), Guangdong (GD), Guangxi (GX), and Fujian (FJ), with 3 replicates of ten fish for each group. The sampling locations of the different populations are shown in Figure 1.

From April to November 2021, *T. blochii* samples were collected from the Sanya breeding base in Hainan Province as experimental materials. 30 healthy fish with body weights of 250.05 ± 13.11 g (4 months of age) and 749.23 ± 24.57 g (9 months of age) were selected. These fish were of the same origin as those in Sanya of Hainan Province described in the previous section, sampled to compliment the aforementioned 497.91 ± 20.61 g (6 months of age) experimental group. In total there were three groups of fish, labeled as 250.05 ± 13.11 g (early stage, ES), 497.91 ± 20.61 g (middle stage, MS), and 749.23 ± 24.57 g (late stage, LS).

In September 2021, *T. blochii* samples with body weights of 512.36 ± 30.02 g were collected from the Sanya breeding base in Hainan Province as experimental materials and divided into two groups according to gender, female and male. Each group of 30 fish was randomly divided into three replicates for the determination of major nutrient components.

The weights of all fish are shown in Supplementary Table S1. After the above samples were collected, they were weighed and immediately put on ice. They were transported back to the laboratory and their back muscles were dissected out and ground before storage in a −20°C freezer and preserved until analysis. All experimental procedures were conducted in accordance with the Guidelines for the Care and Use of Laboratory Animals in China. The Animal Experimentation Ethics Committee of Hainan University approved this protocol (HNUAUCC-2021-00007).

### 2.2. Detection method

According to GB 5009.124–2016 (15), amino acids were determined using ion exchange chromatography involving a ninhydrin column. A standard working solution of mixed amino acids and the solutions of samples for determination were injected into the amino acid analyzer in identical volumes and the concentrations of amino acids in the sample solutions were calculated from the peak areas using the external standard method. Fatty acids were determined according to GB 5009.168–2016 (16) by gas chromatography with a flame hydrogen ion detector (FID). Under chromatographic conditions, the standard determination liquid and sample determination liquid were injected into the gas chromatographer and the chromatographic peak areas were quantified. According to GB 5009.3–2016 (17), moisture content was assayed by oven-drying at an atmospheric pressure of 105°C. Ash content was assayed by high temperature incineration according to GB 5009.4–2016 (18).

### 2.3. Evaluation method

#### 2.3.1. Amino acids

According to the internationally recommended methods from the Food and Agriculture Organization of the United Nations/World Health Organization (FAO/WHO), the AAS (amino acid score), CS (chemical score), and EAAI (essential amino acid index) were calculated. The calculation formulas are as follows (19):

$$\;\mathrm{AAS}=\frac{\mathrm{aa}}{\mathrm{AA}\left(\mathrm{FAO}/\mathrm{WHO}\right)}$$

$$\;\mathrm{CS}=\frac{\mathrm{aa}}{\mathrm{AA}\left(\mathrm{Egg}\right)}$$

$$\mathrm{EAAI}=\sqrt[{n}]{\frac{\mathrm{Tval}}{\mathrm{Sval}}\times \frac{\mathrm{Tleu}}{\mathrm{Sleu}}\times \;\;\;\ldots \;\;\times \frac{\mathrm{Tlys}}{\mathrm{Slys}}}\times 100$$

where, aa is the amino acid content in the sample to be measured [mg/g N]; AA_(FAO/WHO)_ is the content of the same amino acid in the FAO/WHO amino acid scoring model [mg/g N]; AA_(Egg)_ is the content of the same amino acid in the whole egg protein scoring model [mg/gN]; n is the number of amino acids; and T and S are EAA of sample and egg protein, respectively.

In addition, the *F* values of muscle amino acids, namely the ratio of branched AA (BCAA, Val + Ile + Leu) to aromatic AA (AAA, Tyr + Phe), were calculated. The formula is as follows (19):

$$F=\frac{\mathrm{Leu}+\mathrm{Ile}+\mathrm{Val}}{\mathrm{Phe}+\mathrm{Tyr}}$$

#### 2.3.2. Fatty acids

The UFA (unsaturated fatty acid) and SFA (saturated fatty acid) were used to measure the nutritional value of fatty acids. The atherosclerosis index (AI) and thrombosis index (TI) were calculated according to their respective formulas (19), and the effect of fatty acids on human cardiovascular disease was inferred. The lower the AI and TI were, the higher the inhibition activities of atherosclerosis and thrombosis. The polyene index (PI) was calculated according to the formula to reflect the unsaturation of fatty acids:

$\mathrm{AI}=\left(C_{12:0}+C_{14:0}+C_{16:0}\right)/\left(\sum \mathrm{MUFA}+\sum \omega -6+\sum \omega -3\right)$

$\mathrm{TI}=\left(C_{14:0}+C_{16:0}+C_{18:0}\right)/\left[\begin{array}{l} 0.5\times \sum \mathrm{MUFA}+0.5\times \sum \omega -6\\ +3\times \sum \omega -3+\left(\sum \omega -3/\sum \omega -6\right) \end{array}\right]$

$\mathrm{PI}=\left(C_{20:5}+C_{22:6}\right)/C_{16:0}$

### 2.4. Statistical analysis

The experimental data were expressed as means ± SD. Excel and SPSS 26.0 were used for statistical analysis. Independent samples *T*-tests were used to test for significant differences between groups, and *p* < 0.05 was considered statistically significant.

## 3. Results

### 3.1. Muscle compositions of *Trachinotus blochii* in different mariculture areas

**Figure 2:**
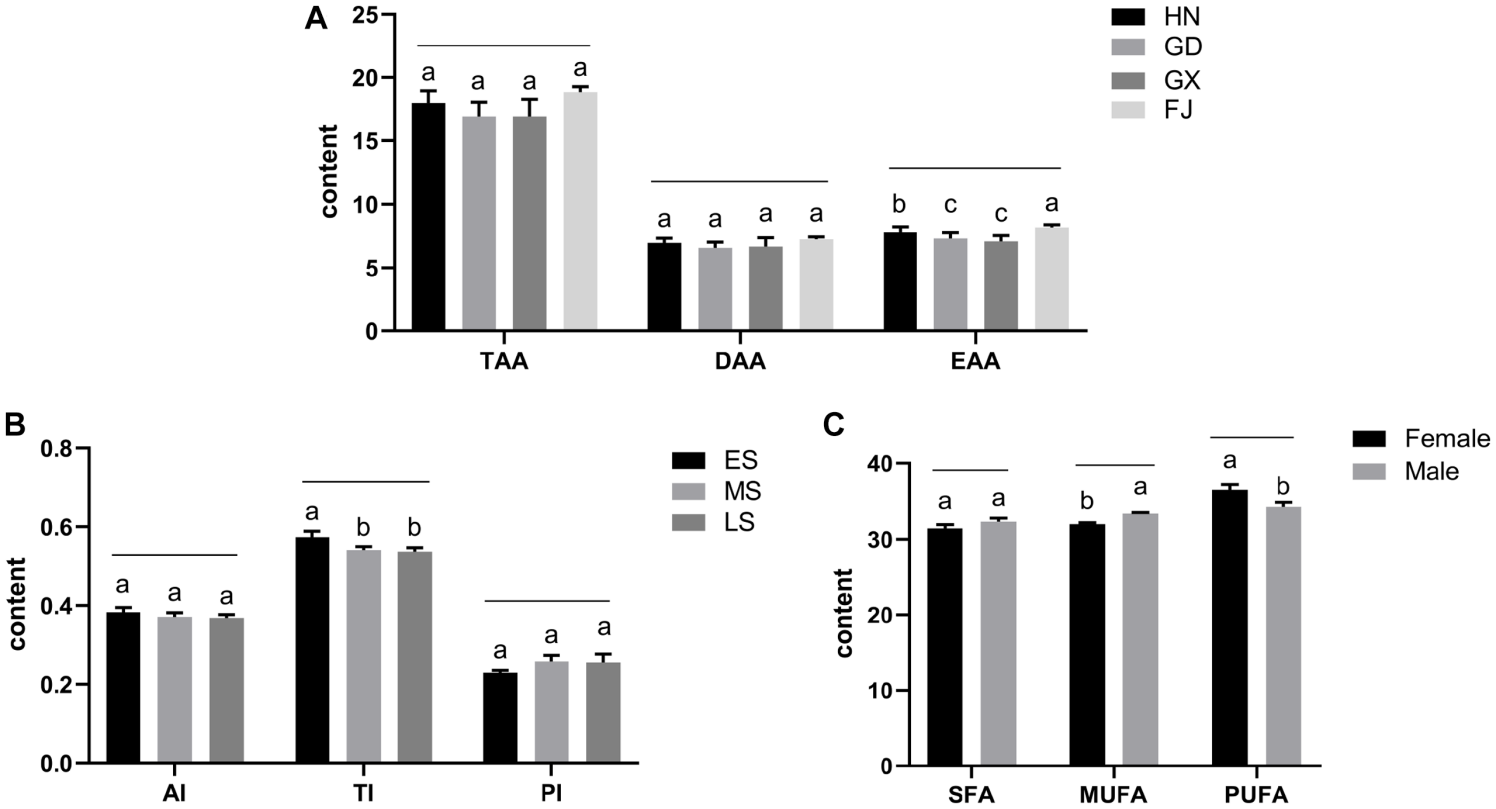
**(A)** Comparison of TAA, DAA and EAA contents in muscle of *T. blochii* in different mariculture areas. **(B)** Comparison of AI, TI, and PI in muscle of *T. blochii* in different growth stages. **(C)** Comparison of SFA, MUFA, and PUFA contents in muscle of female and male *T. blochii*. With the same letter, the difference between them was not significant (*p* > 0.05), without the same letter, the difference was significant (*p* < 0.05).

The nutritional compositions of muscles of *T. blochii* cultured in sea cages in HN, GD, GX, and FJ were analyzed. Amino acid contents of *T. blochii* muscle in each sea culture area are shown in Supplementary Table S2. In total, 16 kinds of amino acids were detected in the muscles of all groups. The contents of total amino acids (TAA) and delicious amino acids (DAA) were higher in the HN and FJ groups. DAAs include Glu, Asp., Phe, Ala, Gly, and Tyr (20, 21). The contents of essential amino acids (EAA) in the GX and GD groups were significantly lower than the HN and FJ groups (*p* < 0.05; Figure 2A). The mean contents of moisture in *T. blochii* muscle were 71.4% ± 1.10% (HN), 73.33% ± 1.36% (GD), 66.33% ± 1.10% (GX) and 69.97% ± 1.60% (FJ), and the GX group was significantly lower than the others (*p* < 0.05). The contents of ash in *T. blochii* muscle were 1.33% ± 0.12% (HN), 1.20% ± 0.17% (GD), 1.07% ± 0.15% (GX) and 1.20%(FJ). In the AAS, CS, and EAAI evaluations, the essential amino acids of the GX group were lower than the other three groups (*p* < 0.05; Table 1).

**Table 1:** Amino AAS, CS evaluation and EAAI of *T. blochii* in different mariculture areas (mg/gN).

Amino acids	FAO/WHO	Egg protein	AAS				CS			
			HN	GD	GX	FJ	HN	GD	GX	FJ
Met+Cys	220	386	0.57 ± 0.02^a^	0.55 ± 0.07^a^	0.45 ± 0.05^b^	0.58 ± 0.05^a^	0.32 ± 0.01^a^	0.31 ± 0.04^a^	0.25 ± 0.03^b^	0.33 ± 0.03^a^
Val	310	410	0.67 ± 0.02^a^	0.67 ± 0.03^a^	0.52 ± 0.04^b^	0.67 ± 0.04^a^	0.50 ± 0.01^a^	0.51 ± 0.02^a^	0.39 ± 0.03^b^	0.51 ± 0.04^a^
Ile	250	331	0.74 ± 0.01^a^	0.75 ± 0.03^a^	0.58 ± 0.05^b^	0.75 ± 0.06^a^	0.56 ± 0.01^a^	0.57 ± 0.02^a^	0.44 ± 0.04^b^	0.57 ± 0.05^a^
Leu	440	534	0.76 ± 0.02^a^	0.76 ± 0.03^a^	0.58 ± 0.05^b^	0.75 ± 0.06^a^	0.62 ± 0.01^a^	0.63 ± 0.03^a^	0.48 ± 0.04^b^	0.62 ± 0.05^a^
Phe + Tyr	380	565	0.82 ± 0.01^a^	0.82 ± 0.02^a^	0.64 ± 0.03^b^	0.81 ± 0.04^a^	0.55 ± 0.01^a^	0.55 ± 0.02^a^	0.43 ± 0.02^b^	0.55 ± 0.02^a^
Thr	250	292	0.78 ± 0.02^a^	0.79 ± 0.03^a^	0.61 ± 0.06^b^	0.78 ± 0.06^a^	0.67 ± 0.02^a^	0.67 ± 0.03^a^	0.52 ± 0.05^b^	0.67 ± 0.05^a^
Lys	340	441	1.15 ± 0.02^a^	1.17 ± 0.06^a^	0.88 ± 0.08^b^	1.16 ± 0.09^a^	0.88 ± 0.02^a^	0.90 ± 0.02^a^	0.68 ± 0.06^b^	0.89 ± 0.07^a^
EAAI			56.51 ± 1.00^a^		56.72 ± 2.66^a^		43.88 ± 3.51^b^		56.91 ± 3.93^a^	

^a,b^With the same letter, the difference between the group was not significant (*p* > 0.05), without the same letter, the difference was significant (*p* < 0.05).

**Figure 3:**
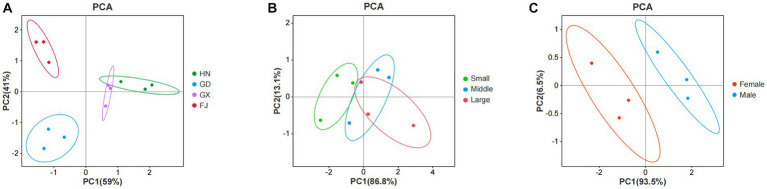
Principal Component Analysis (PCA) of SFA, MUFA, and PUFA datasets in different mariculture areas **(A)**, growth stages **(B)**, and genders **(C)**.

The monounsaturated fatty acid (MUFA) content was significantly higher in the FJ group than all other groups (*p* < 0.05), it was lowest in the HN group, and the GD group was significantly higher than the HN and GX groups (*p* < 0.05; Supplementary Table S3). The contents of polyunsaturated fatty acids (PUFA) were 36.68% ± 0.94% (HN), 30.72% ± 0.69% (GD), 34.7% ± 0.28% (GX), and 31.43% ± 0.25% (FJ). The PCA analysis of the SFA, MUFA, and PUFA datasets in the 4 mariculture areas is shown in Figure 3A. The AI was lowest in the FJ group and TI was lowest in the HN group. The PI was highest in the HN group, at 0.26 ± 0.02, and lowest in the GD group, at 0.17 ± 0.01.

### 3.2. Muscle compositions of *Trachinotus blochii* in different growth stages

The muscle nutritional compositions of cultured *T. blochii* were analyzed in three different growth stages, including 250 ± 19.37 g (ES), 500 ± 28.36 g (MS), and 750 ± 29.97 g (LS). According to Supplementary Table S4, a total of 16 amino acids were detected in the muscle, including 7 EAAs, 1 CEAA, and 8 NEAAs, which was consistent with the results of the *T. blochii* raised in different areas. The content of TAA increased with the growth in *T. blochii*, but the difference was not significant (*p* > 0.05). The content of DAA also increased gradually with the growth of *T. blochii*. Moisture made up the majority of *T. blochii* muscle tissue, with averages of 70.57% ± 2.56% (ES), 71.4% ± 1.1% (MS), and 71.17% ± 1.22% (LS) measured in this experiment. The ash content increased with growth in *T. blochii* (*p* > 0.05). The AAS, CS, and EAAI increased gradually in all groups during the growth of *T. blochii*, indicating that the nutritional value of amino acids in muscle of cultured *T. blochii* gradually increased with growth in *T. blochii* (Table 2).

**Table 2:** Amino acids AAS, CS evaluation, and EAAI of *T. blochii* with different sizes (mg/gN).

Amino acids	FAO/WHO	Egg protein	AAS			CS		
			Early stage (250 g)	Middle stage(500 g)	Late stage(750 g)	Early stage (250 g)	Middle stage(500 g)	Late stage(750 g)
Met+Cys	220	386	0.56 ± 0.04^a^	0.57 ± 0.02^a^	0.58 ± 0.02^a^	0.32 ± 0.01^a^	0.32 ± 0.02^a^	0.33 ± 0.02^a^
Val	310	410	0.65 ± 0.04^a^	0.67 ± 0.02^a^	0.69 ± 0.03^a^	0.49 ± 0.01^a^	0.51 ± 0.02^a^	0.52 ± 0.02^a^
Ile	250	331	0.72 ± 0.05^a^	0.74 ± 0.01^a^	0.76 ± 0.03^a^	0.55 ± 0.01^a^	0.56 ± 0.03^a^	0.58 ± 0.02^a^
Leu	440	534	0.73 ± 0.06^a^	0.75 ± 0.02^a^	0.76 ± 0.03^a^	0.60 ± 0.01^a^	0.62 ± 0.03^a^	0.60 ± 0.03^a^
Phe + Tyr	380	565	0.79 ± 0.06^a^	0.82 ± 0.02^a^	0.84 ± 0.03^a^	0.53 ± 0.01^a^	0.55 ± 0.03^a^	0.57 ± 0.02^a^
Thr	250	292	0.76 ± 0.05^a^	0.78 ± 0.02^a^	0.79 ± 0.03^a^	0.65 ± 0.02^a^	0.67 ± 0.03^a^	0.68 ± 0.03^a^
Lys	340	441	1.12 ± 0.09^a^	1.14 ± 0.02^a^	1.17 ± 0.05^a^	0.87 ± 0.02^a^	0.88 ± 0.04^a^	0.90 ± 0.04^a^
EAAI			55.09 ± 1.10^b^		56.51 ± 2.19^ab^		57.78 ± 2.19^a^	

^a,b^With the same letter, the difference between the group was not significant (*p* > 0.05), without the same letter, the difference was significant (*p* < 0.05).

Fatty acid analysis identified 29 fatty acids in *T. blochii* muscle, including 12 SFA, 7 MUFA, and 10 PUFA (Supplementary Table S5). The PCA analysis of the SFA, MUFA, and PUFA datasets of *T. blochii* at different growth stage is shown in Figure 3B. The PUFA content was highest in the LS group, at 37.006 ± 1.19 g /100 g, followed by the MS group, at 36.676 ± 0.94 g /100 g, and lowest in the ES group, at 35.15 ± 0.73 g /100 g, revealing a gradually increasing tendency with growth of *T. blochii*. The PI was highest in the MS group, followed by the LS group, and finally the ES group with the lowest value. The AI and TI decreased with growth in *T. blochii* (Figure 2B).

### 3.3. Muscle compositions of female and male *Trachinotus blochii*

According to Supplementary Table S6, the TAA contents of the two groups were 18.2 ± 0.5 g /100 g (♀) and 18.13 ± 1.25 g /100 g (♂). The EAA values were 8.07 ± 0.2 g /100 g (♀) and 7.98 ± 0.49 g /100 g (♂), higher in females than males (*p* > 0.05). The mean values of EAA/TAA were 44.36% and 44.03%, and the mean values of EAA/NEAA were 79.72% and 78.67% for females and males, respectively, and while females were slightly higher, the differences were not significant (*p* > 0.05). According to the FAO/WHO ratio, the muscle protein of cultured *T. blochii* was high quality. The branch/aromatic values of amino acids were 2.299 ± 0.002 (♀) and 2.368 ± 0.078 (♂), indicating both male and female *T. blochii* had liver protecting qualities. The moisture content was slightly higher in female *T. blochii* than in males, but the differences were not significant (*p* > 0.05). The AAS, CS, and EAAI values showed that the amino acid content of female pompanos were slightly higher than those of males, but the difference was not significant (*p* > 0.05; Table 3).

**Table 3:** Amino acids AAS, CS evaluation, and EAAI of male and female *T. blochii* (mg/gN).

Amino acids	FAO/WHO	Egg protein	AAS		CS	
			Female	Male	Female	Male
Met+Cys	220	386	0.62 ± 0.01^a^	0.35 ± 0.01^b^	0.54 ± 0.06^a^	0.31 ± 0.03^b^
Val	310	410	0.72 ± 0.02^a^	0.54 ± 0.01^a^	0.67 ± 0.10^a^	0.51 ± 0.07^a^
Ile	250	331	0.80 ± 0.20^a^	0.61 ± 0.02^a^	0.73 ± 0.10a	0.55 ± 0.08^a^
Leu	440	534	0.81 ± 0.20^a^	0.66 ± 0.01^a^	0.71 ± 0.80^a^	0.58 ± 0.07^a^
Phe + Tyr	380	565	0.89 ± 0.20^a^	0.60 ± 0.01^b^	0.78 ± 0.10^a^	0.52 ± 0.05^b^
Thr	250	292	0.84 ± 0.02^a^	0.72 ± 0.01^a^	0.73 ± 0.08^a^	0.63 ± 0.07^a^
Lys	340	441	1.24 ± 0.02^a^	0.96 ± 0.02^b^	1.10 ± 0.12^a^	0.85 ± 0.09^b^
EAAI			61.14 ± 1.26^a^		54.35 ± 6.41^b^	

^a,b^With the same letter, the difference between the group was not significant (*p* > 0.05), without the same letter, the difference was significant (*p* < 0.05).

There were 29 fatty acids in the *T. blochii* muscle of both males and females, which was consistent with the previous results (Supplementary Table S7). The SFA contents of males was higher than females (*p* > 0.05) and the PUFA content was significantly higher in females than males (*p* < 0.05; Figure 2C). The PCA analysis of the SFA, MUFA, and PUFA datasets in male and female *T. blochii* is shown in Figure 3C. The analysis showed that the AI and TI in female *T. blochii* were lower than in males, and the PI of females was higher than in males, but there was no significant difference (*p* > 0.05).

## 4. Discussion

### 4.1. Amino acid composition analysis and evaluation

In dietary amino acids, EAAs are generally the most important to the general public. This study showed that the EAA content in the GX and GD groups were significantly lower than those in the HN and FJ groups (*p* < 0.05). Similarly, the amino acid contents were compared in *Megalobrama terminalis* among three geographical populations (22), *Larimichthys crocea* from 4 kinds of sources (23), and *Larimichthys polyactis* from four different localities (24) were measured, and all found significant differences in amino acid contents. In combination with our results, the contents of EAAs in muscle of *T. blochii* varied greatly among different mariculture areas, even though the fish were all of the same origin, had the same growth period, and were fed the same diet. These results indicated that the nutrient composition of fish is closely related to their aquatic environment and naturally available biological feed. According to the AAS and CS scoring criteria, the AAS score of Lys was greater than 1 in fish of all groups except GX, indicating that the Lys in *T. blochii* muscle exceeded the FAO/WHO and egg protein standards. Lys is an essential amino acid in the human body and can boost the development of the human body (25). Normal human diets are high in grains and contain relatively low Lys, so increasing the intake of Lys-rich fish may provide much needed Lys supplement (26).

The contents of TAA, EAA, and DAA increased with the growth of *T. blochii* at different growth stages, but the differences were not significant (*p* > 0.05). This was consistent with the results of a study on *Pangasius sutchi* (27). It has been demonstrated that, the more abundant the essential amino acids, the higher the nutritional value of the fish (28), which means that *T. blochii* in the later growth stages are more nutritious and can better meet the essential amino acid requirements of humans. The amino acid AAS, CS, and EAAI in each group gradually increased with the growth of *T. blochii*. The higher the EAAI index, the more balanced the amino acid composition, indicating better protein quality and higher utilization rates (29). This means that the muscles of larger *T. blochii* have greater health benefits for humans.

There were no significant differences in TAA, DAA, EAA, NEAA, and EAA/NEAA between male and female *T. blochii* (*p* > 0.05). Similar results were observed in muscle nutrition analyses of male and female *Hippocampus Erectus* (30) and *Acrosscheilus wenchowensis* (31). However, our results were different from those observed in *T. ovatus* (32). This may have been related to the differences among species and sizes of fish and because the sexual dimorphism between males and females becomes more pronounced in the later growth stages, which will produce further differences in their nutritional compositions. Nevertheless, The EAAI of female *T. blochii* was higher than that of males, indicating that the nutritional value of females was higher than that of males, which was consistent with the observations made in *Centropristis striata* (33) and *Trachinotus ovatus* (32). This may be related to differences in growth rates, gonadal development requirements, and energy consumption patterns in males and females, as well as diet intake and conversion rates. Similarly, our previous work has shown that the testes developed faster than ovaries in *T. blochii* before the age of 12 months (unpublished), which may mean that more nutrients are used during male development and energy expenditure, resulting in decreased muscle quality, but this would need to be confirmed with further study.

### 4.2. Fatty acid composition analysis and evaluation

The nutritional value of fish is also related to the variety, content, and composition of fatty acids. For the general public, the most desirable dietary fat composition is as follows: S: M: P (SFA: MUFA: PUFA) of 1:1:1 (34). The fatty acid composition of *T. blochii* basically met this requirement. According to the evaluated AI, TI, and PI values of fatty acids and in combination with the UFA values, it was clear that *T. blochii* cultured in HN and FJ had higher unsaturated fatty acids and higher inhibition of atherosclerosis and thrombosis activity. These attributes indicated that daily consumption could improve cardiovascular function in humans (35). The differences among the locations may be related to differences in the species and distributions of macrobenthic animals which represent an important natural food source for fish like *T. blochii*. Indeed, a study of benthic environments in different aquaculture areas observed clear differences in the distributions of these naturally occurring benthic organisms (36). Furthermore, the differences in fatty acid contents of *T. blochii* raised in different mariculture areas with differences in food availability might be related to the local environmental conditions and their influence on ecological communities. At different growth stages, SFA content in the muscle of *T. blochii* was seen to decrease with growth, while MUFA content first decreased and then increased with increasing size and PUFA content increased with increasing size. The fatty acid trends observed in this study were similar to those of other species, including *Ammodytes personatus* (37), *Erythroculter ilishaeformis* (38), and *Hapalogenys mucronatus* (39). PUFA has obvious lipid-lowering, hypotensive, anti-tumor, and immunomodulatory effects on human body (40, 41), which means that late growth stage *T. blochii* should have high nutritional value and health benefits. Some studies have indicated that the changes in fatty acid content may be associated with the development of gonads, during which fish accumulate PUFA to meet their reproductive needs (42). Therefore, we speculated that the accumulation of PUFA in *T. blochii* may be used for gonad development. In this study, PUFA in females was significantly higher than in males (*p* < 0.05), which may have been because, during development of the testis, lipids were mainly supplied from the internal fat stored in the muscle. The AI and TI of female *T. blochii* were lower than those of males (*p* > 0.05), indicating that the regular consumption of *T. blochii* should have health benefits, and the nutritional value of female *T. blochii* is slightly higher than that of male *T. blochii*.

*T. blochii* contained an abundance of amino acids and fatty acids. The TAA content of *T. blochii* muscle was 17.93%–18.57%. The higher the amino acid content and diversity, the higher the nutritional value of the muscle (28). The composition and content of DAA determine the taste and flavor of the fish. Here, the ratio of DAA/TAA ranged within 38.56%–39.37%, which was higher than in *Pseudobagrus ussuriensis* (25.42%) and *Silurus glanis Linnaeus* (34.18%) (43), indicating that *T. blochii* tasted better. Furthermore, the EAAI of *T. blochii* ranged from 54.35 to 61.14, which was significantly higher than that of *Megalobrama amblycephala* (46.96%) (44). A higher EAAI index indicates a more balanced amino acid composition and higher potential utilization efficiency (45). Therefore, *T. blochii* muscle should be readily absorbed by the human body. The fatty acid composition of muscles is also an important factor affecting quality and flavor (46). The PUFA content of *T. blochii* accounted for 30%–37% of the fatty acid content, which was notably higher than those of *Oncorhynchus keta* (14.7%) (47) and *Oncorhynchus kisutch* (28.78%) (48). This study, which assessed different mariculture areas, different growth stages, and different genders, found that the HN and FJ groups, the later growth stages, and the female *T. blochii* had the highest nutritional values.

## 5. Conclusion

This study has revealed that *T. blochii* is a fish with high nutritional value. The UFAs and EAAs, which are beneficial to human health, were highest in the muscle of *T. blochii* cultured in HN and FJ. Furthermore, *T. blochii* in the later growth stages had higher nutritional and health values. Similarly, the nutritional value of female *T. blochii* was slightly higher than that of males. In conclusion, *T. blochii* is a high-quality species suitable for large-scale breeding and human consumption should be encouraged. To maximize marketability, all-female breeding should be researched as a future breeding direction.

## Data availability statement

The raw data supporting the conclusions of this article will be made available by the authors, without undue reservation.

## Ethics statement

The animal study was reviewed and approved by the Animal Experimentation Ethics Committee of Hainan University.

## Author contributions

LS: writing – original draft, writing – review, and editing. FS: conceptualization, methodology, formal analysis, and funding acquisition. SX: data analysis and writing. WZ: sample collection. YL: sample collection. KZ: sample collection. JS: visualization. JL: conceptualization, methodology, supervision, project administration, sample acquisition, and article revision. All authors contributed to the article and approved the submitted version.

## Funding

This work was supported by the Hainan Provincial Natural Science Foundation of China (322QN236), the National Natural Science Foundation of China (32160862), the project of Hainan Yazhou Bay Seed Laboratory (B21HJ0105), and the Initial Fund from Hainan University for R&D, KYQD (ZR)-2013.

## Acknowledgments

The authors were grateful to all the laboratory members for their continuous technical advice and helpful discussion.

## Conflict of interest

The authors declare that the research was conducted in the absence of any commercial or financial relationships that could be construed as a potential conflict of interest.

## Publisher’s note

All claims expressed in this article are solely those of the authors and do not necessarily represent those of their affiliated organizations, or those of the publisher, the editors and the reviewers. Any product that may be evaluated in this article, or claim that may be made by its manufacturer, is not guaranteed or endorsed by the publisher.

## Supplementary material

The Supplementary material for this article can be found online at: https://www.frontiersin.org/articles/10.3389/fnut.2023.1148687/full#supplementary-material
